# Fabrication of 3D HierarchicalSphericalHoneycomb-Like Nd_2_O_3_/Co_3_O_4_/Graphene/Nickel Foam Composite Electrode Material for High-Performance Supercapacitors

**DOI:** 10.3390/ma16041694

**Published:** 2023-02-17

**Authors:** Huihui Liang, Shasha Wang, Shixiang Lu, Wenguo Xu, Min Zhou

**Affiliations:** 1 School of Chemistry and Chemical Engineering, Beijing Institute of Technology, Beijing 100081, China; 2 School of Chemical Science and Technology, Yunnan University, Kunming 650091, China

**Keywords:** graphene, Nd_2_O_3_, Co_3_O_4_, nickel foam, supercapacitor

## Abstract

A 3D hierarchical spherical honeycomb-like composite electrode materialof neodymium oxide (Nd_2_O_3_), cobalt tetraoxide (Co_3_O_4_), and reduced graphene oxide (rGO) on nickel foam (named as Nd_2_O_3_/Co_3_O_4_/rGO/NF) were successfully fabricated by combining the hydrothermal synthesis method and the annealing process. Nickel foam with a three-dimensional spatial structure was used as the growth substrate without the use of any adhesives. The Nd_2_O_3_/Co_3_O_4_/rGO/NF composite has outstanding electrochemical performance and can be used directly as an electrode material for supercapacitors (SCs). By taking advantage of the large specific surface area of the electrode material, it effectively slows down the volume expansion of the active material caused by repeated charging and discharging processes, improves the electrode performance in terms of electrical conductivity, and significantly shortens the electron and ion transport paths. At a 1 A/g current density, the specific capacitance reaches a maximum value of 3359.6 F/g. A specific capacitance of 440.4 F/g with a current density of 0.5A/g is still possible from the built symmetric SCs. The capacitance retention rate is still 95.7% after 30,000 cycles of testing at a high current density of 10 A/g, and the energy density is 88.1 Wh/kg at a power density of 300 W/kg. The outcomes of the experiment demonstrate the significant potential and opportunity for this composite material to be used as an electrode material for SCs.

## 1. Introduction

With the development of the economy and society, people’s dependence on energy has been increasingsince about the Industrial Revolution. These energy sources cannot be separated from the extraction and consumption of fossil fuels [1,2], and it is sure to happen that rapid economic development has brought serious environmental pollution at the same time [3,4]. However, as sustainable energy sources such aswind and solar are easily impacted by location and time, urgent research and development into novel energy storage technologies are required [5]. Supercapacitors (SCs) are energy storage devices that outperform batteries in terms of lifecycle, speed of charging and discharging, power, safety, non-toxicity, and cleanliness [6]. The current aim of SC research is to increase the energy density and capacitance of the materials utilized [7,8]. According to their methodology of pumped storage, SCs can be split into double-layer capacitors, pseudocapacitor capacitors, and hybrid capacitors. The portion of charge storage capacity of the double-layer capacitors is based primarily on the electrode material’scapacity to absorb ions on the surface [9,10]. The charge storage method in pseudocapacitors is accomplished via a quick and reversible Faraday redox reaction between the electrode material on the electrode surface and the ions in the electrolyte. As a result, the large specific surface area of the electrode material surface structure can also more effectively provide ions to the electrode. Both of these procedures result in the charge storage principle of hybrid SC.

Multi-component metal sulfides and oxides are the most prevalent pseudocapacitive electrode materials because they can generate both double-layer capacitance and pseudocapacitance through electrochemical processes during the charge storage process [11]. The successful fabrication of such electrode materials opens up a whole new horizon for electrode materials for SCs, not only by modifying the ratio between different metal elements to seek greater potential window, conductivity, and other aspects of electrode materials, but also by thinking about compounding with other carbon-based materials to make full use of the synergy between different materials, to complement each other’s strengths, and to build electrode materials for SCs that are more robust and durable [12,13]. Researchers recently created brand-new electrode materials from inorganic rare-earth elements in an effort to better the electrochemical properties of electrode materials [14]. Rare earth elements are highly valued for their exceptional physicochemical qualities, which are derived from their unique 4f electron configuration and used in optics, catalysts, and energy devices [15,16]. LaFeO_3_ (LF) chalcogenides were created by doping Mn and Nd during hydrothermal synthesis to produce La_0.8_Nd_0.2_Fe_0.8_Mn_0.2_ (LNFM) [17]. When the scan rate was 50 mV/s, the specific capacitance of the electrode could still reach 158 F/g, which was much higher than that of the pure LF sample and the single-doped LF. The capacity retention of the composite material was also discovered to be 92.4% at 10,000 cycles (85.37% for the LNFM sample) during the research on the cycling stability of the electrode material. Additionally, research has shown that doping cobalt trioxide with rare-earth-based elements such as lanthanum, neodymium, gadolinium, and samarium improved the performance of the materials to varying degrees. Rare earth elements were discovered to be involved in lowering charge transfer resistance during doping, and this structural defect caused by doping can be seen clearly by scanning electron microscope (SEM) while also shortening the path that electrolyte ions take during diffusion [18]. Still, there are notmany studies on the use of lanthanide rare earth metals in SCs.

Here, Nd_2_O_3_/Co_3_O_4_/reduced graphene oxide (rGO) with a three-dimensional porous honeycomb spherical structure with a high specific surface area of 107.6246 m^2^/g and an average pore size of about 12 nm were successfully prepared experimentally using a simple one-step hydrothermal synthesis and annealing. When the Nd_2_O_3_/Co_3_O_4_/rGO/NF composite electrode was implemented as the electrode material with a current density of 1 A/g, its specific capacitance was 3359.6 F/g. In addition, the prepared electrodes can be directly assembled into symmetrical SCs with a specific capacitance of 440.4 F/g at a capacitance density of 0.5 A/g and a capacitance retention rate of 95.7% (30,000 cycles) even at a high current density of 10 A/g, making Nd_2_O_3_/Co_3_O_4_/rGO/NF an SC electrode material with great application prospects.

## 2. Materials and Methods

### 2.1. Materials

The original graphite powder, concentrated HCl (36%), concentrated H_2_SO_4_ (98%), concentrated KMnO_4_ (99%), ethanol (99.3%), hydrogen peroxide (30%wt), acetone (99.6%), and potassium hydroxide (KOH) were all purchased from Beijing Fine Chemicals Co. Ltd. (Beijing, China) Nickel foam (NF) is made available by Kunshan Tonghui Electronic Technology Co. Ltd. (Kunshan, China) Shanghai Maclean Biochemical Co. Ltd. (Shanghai, China) prepared urea (CH_4_N_2_O), cobalt nitrate hexahydrate (Co(NO_3_)_2_‣6H_2_O), and neodymium acetate pentahydrate (Nd(CH_3_COO)_3_‣5H_2_O). All of the chemical reagents used in the experiments were analytically pure and employed in accordance with the set usage guidelines.

### 2.2. Preparation of Nd_2_O_3_/Co_3_O_4_/rGO/NF Composite Electrode

#### 2.2.1. Pretreatment of Conductive Substrate NF

The NFs (20 mm × 10 mm × 1 mm) were manually cut beforehand, washed with acetone, ethanol, and deionized water in turn for 30 min in an ultrasonic water bath, and then dried in an oven at 60 °C for 12 h to get rid of any potential contaminants.

#### 2.2.2. Synthesis of Nd_2_O_3_/Co_3_O_4_/rGO/NF Precursors

The GO used in the experiments was obtained by using graphite powder as raw material and prepared by using the modified Hummer method [19]. A total of 0.6 mmol (0.175 g) Co(NO_3_)_2_‣6H_2_O, 0.6 mmol (0.247 g) Nd(CH_3_COO)_3_‣5H_2_O and 1 mmol (0.060 g) urea were added to 7.5 mL deionized water to dissolve it fully and then continued to stir magnetically for 40 min, then add 7.5 mL 2 mg/mL GO homogeneous suspension that has been sonicated for 2 h and continue to stir by magnetic force for 40 min to ensure that the black solution A is mixed well. In a volume of 25 mL PTFE-lined reactor, the pretreated nickel foam was mixed with solution A and placed in an oven for 12 h at 140 °C. Then it was rinsed several times with deionized water and anhydrous ethanol and dried in the oven (90 °C, 4 h), and set aside.

#### 2.2.3. Synthesis of Nd_2_O_3_/Co_3_O_4_/rGO/NF

**Figure 1:**
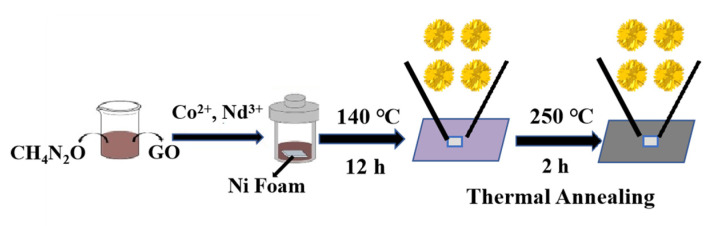
The preparation process of Nd_2_O_3_/Co_3_O_4_/rGO/NF composite electrode.

The precursor samples prepared above were calcined in a muffle furnace, and annealed at 250 °C for 2 h. After cooling to room temperature, the surface of NF was observed to change from light purple to black, and the composite electrode of Nd_2_O_3_/Co_3_O_4_/rGO/NF and Nd_2_O_3_/Co_3_O_4_/rGO was obtained with an average mass of 1.50 ± 0.11 mg/cm^2^ loaded on NF. The Co_3_O_4_/rGO/NF composite electrode was synthesized as shown in Figure 1. The experimental procedures for the preparation of Nd_2_O_3_/Co_3_O_4_/rGO, Nd_2_O_3_/rGO/NF, Co_3_O_4_/rGO/NF and rGO/NF are shown in supplementary S1–S4.

### 2.3. Structural Characterization

The constituent structures of the materials were characterized and analyzed by an x-ray electron spectrometer (XPS, model PHI 5300, Physical Electronics, Chanhassen, MN, USA) and a Bruker D8 Advance x-ray powder diffractometer (XRD, 2θ = 10°~80°) equipped with Cu K*_α_* (λ = 0.15418 nm). The surface morphological structure of the sample materials was visualized using a scanning electron microscope (SEM, Quanta 600, FEI, Rock Hill, SC, USA) with its own energy spectrometer (EDS, Oxford, Gemini 300), a high-resolution transmission electron microscope (HRTEM) and a transmission electron microscope (TEM, JEM-2100, JEOL, Tokyo, Japan). A Renishaw InVia confocal Raman spectrometer, a Leica DMLM microscope, and an argon ion laser (514.5 nm, model Stellar-REN, Modu-Laser, Centerville, UT, USA) as the excitation source were used to capture the Raman spectra.

### 2.4. Electrochemical Characterization

The produced electrodes were electrochemically tested on an electrochemical workstation (CHI 760E, CH Instruments, Bee Cave, TX, USA) using a standard three-electrode system and a potassium hydroxide solution at a concentration of 6 mol/L as the electrolyte. The prepared electrode served as the working electrode during the test, with the saturated calomel electrode serving as the reference electrode and the platinum electrode performing as the counter electrode.

#### 2.4.1. Cyclic Voltammetry (CV)

To illustrate the variation in electrochemical performance between various electrodes, the CV curves of various sample electrodes were measured and compared. The charge (capacity, in C/g) can be calculated could be calculated from the CV curves, using the following Equation (1) [20].

(1) $$C=\frac{1}{E\nu m}\int_{V_{1}}^{V_{2}}idV$$

where E is the potential window in this equation(E = V_2_ − V_1_, where V_2_ and V_1_ are the potential window’s edges), ν  is the scan rate, i is the discharge (or charge) current, and dV is the infinitesimal potential change.

#### 2.4.2. Constant Current Charge and Discharge (GCD)

The specific capacitance of the corresponding electrodes was determined by measuring and comparing the GCD curves of various sample electrodes at current densities of 1~10 A/g and voltage windows of −0.1~0.4 V. The specific capacitance of the three-electrode system can also be calculated and is listed in Equation (2) [21].

(2) $$C=\frac{\;\left(I\times \Delta t\right)}{\left(m\times \Delta V\right)}$$

Equation (3) is used to determine the energy density of the electrodes (E, Wh/kg) and Equation (4) is used to determine the power density (P, W/kg) in order to define the symmetric SC [22].

(3) $$E=\frac{\left(C\times \Delta V^{2}\right)}{\left(2\times 3.6\right)\;}$$

(4) $$P=\frac{\left(3600\times E\right)}{\Delta t\;}$$

where C stands for the specific capacitance of electrode, I exemplifies the constant discharge current, t exemplifies the discharge time, m exemplifies the mass of the active material on the electrode, and V (V) exemplifies the total voltage window.

#### 2.4.3. Electrochemical Impedance Spectroscopy (EIS)

With the use of EIS, the electrode interfacial resistance and mass transfer resistance are qualitatively assessed, and the causes of the enhanced electrochemical performance are looked into.

At open circuit voltage amplitude of 5 mV, EIS is measured in the frequency range of 100 kHz to 0.01 Hz.

#### 2.4.4. Test of Cycling Stability

Using a constant current charge/discharge cycle test, the electrochemical stability of the Nd_2_O_3_/Co_3_O_4_/rGO/NF composite electrode was assessed in this study. The outstanding electrochemical performance and cycling stability of the composite electrode were confirmed after 30,000 cycles of the stability test.

## 3. Results and Discussion

### 3.1. Analyses of Morphology and Structure

**Figure 2:**
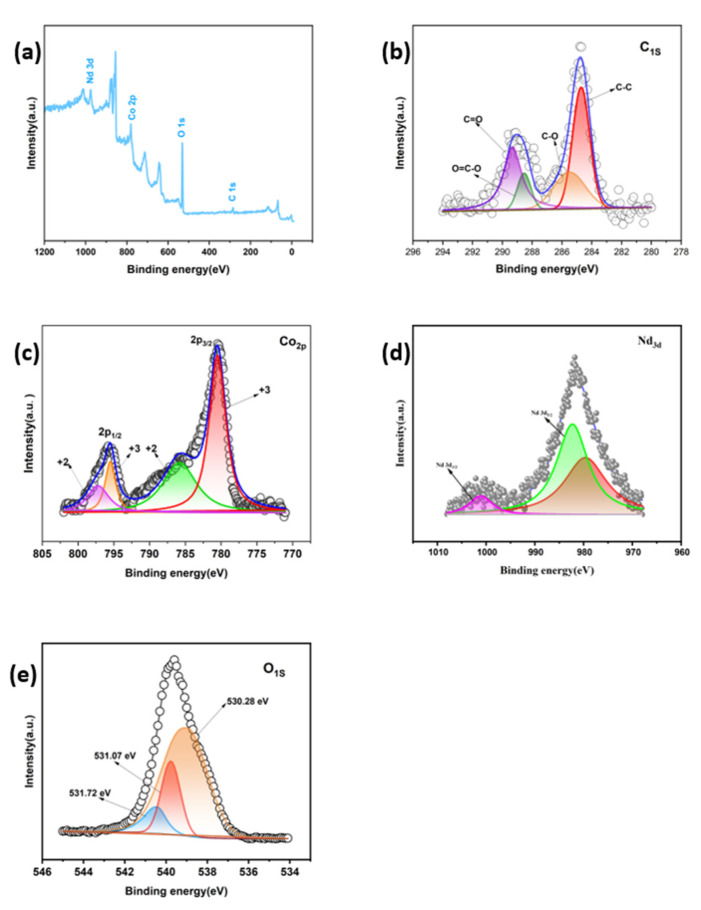
(**a**) XPS spectra of the Nd_2_O_3_/Co_3_O_4_/rGO composite electrode material; High-resolution XPS spectra of (**b**) C1s; (**c**) Co2p; (**d**) Nd3d; and (**e**) O1s of the Nd_2_O_3_/Co_3_O_4_/rGO composite electrode material.

The produced material was measured and examined using XPS in the scanning range of 0~1000 V in order to investigate and describe the elemental composition and chemical state of the material. In the necessary scanning range, it was uncovered that four elements, Nd, Co, C, and O, were for certain as shown in Figure 2a. The presence of three peaks can be seen in the C1s high-resolution spectrum as shown in Figure 2b, with the strongest peak at 284.8 eV indicating the presence of C-C bonds and the other relatively weak peaks at 285.3 eV and 289.2 eV indicating the presence of C-O and C=O bonds, respectively. It can be inferred from the foregoing analysis that GO was successfully reduced in the experiment. Additionally, the high-resolution O1s spectra as shown in Figure 2e demonstrate that the peak at 529.6 eV is associated with the presence of the Co-O bond in the material [23], the peak at 531.6 eV is attributable to lattice oxygen (Nd/O bond) [24], and the remaining peak at 530.8 eV is linked to the material capability to adsorb oxygen molecules [25]. Various positions of peaks can be seen in the high-resolution spectrum of the element Co, where the peak positions at 780.1 eV and 795.3 eV correspond to the typical peaks of Co2p_3/2_ and Co2p_1/2_ of Co_3_O_4_ in the material, respectively, as shown in Figure 2c. Additionally, it can be seen that the Co2p_3/2_ peak has a satellite peak at 781.8.5 eV/780.1 eV and the Co2p_1/2_ peak has a satellite peak at 796.7 eV/795.3 eV, indicating that there is a simultaneous presence of Co^2+^ and Co^3+^ in the material [26]. The magnitude of the binding energy difference between the two peak positions is about 15.2 eV. This phenomenon indicates the presence of Co(II)/Co(III) in the sample. The high-resolution XPS pattern of Nd3d in the image as shown in Figure 2d has two distinct peaks at 982.3 eV and 1001.4 eV [27], which are caused by the spin splitting of Nd3d orbitals into Nd3d_5/2_ and Nd3d_3/2_ orbitals with an energy difference of 19.1 eV [28]. This is evidence that Nd_2_O_3_ was successfully prepared for the experiment.

**Figure 3:**
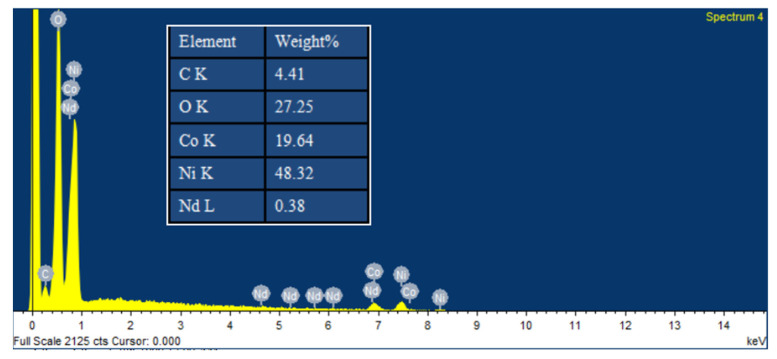
EDS of Nd_2_O_3_/Co_3_O_4_/rGO/NF.

X-ray energy spectrometry (EDS) was used to identify the elemental makeup of the Nd_2_O_3_/Co_3_O_4_/rGO/NF composite electrode. There is no doubt that the manufactured composite electrode material is made up of the five components, O, C, Co, Ni, and Nd, as indicated in Figure 3. EDS spectroscopy can be used to estimate the required ratio of each element on the surface of the composite electrode material. The mass percentages of Co and Nd are 19.64% and 0.38%, respectively. Element C corresponds to the presence of graphene in the material, while elements Nd and Co correspond to the presence of Nd_2_O_3_and Co_3_O_4_, respectively, which were successfully prepared and loaded onto the surface of graphene. This result provides a more precise confirmation that the NF substrate has been successfully loaded with Nd_2_O_3_ and Co_3_O_4_. Additionally, elemental mapping analysis can be used to investigate the elemental distribution in Nd_2_O_3_/Co_3_O_4_/rGO/NF. The elements, C, O, Co, Ni, and Nd, had a homogeneous distribution on the electrode, as illustrated in Figure S1, and there were no impurities observed.

**Figure 4:**
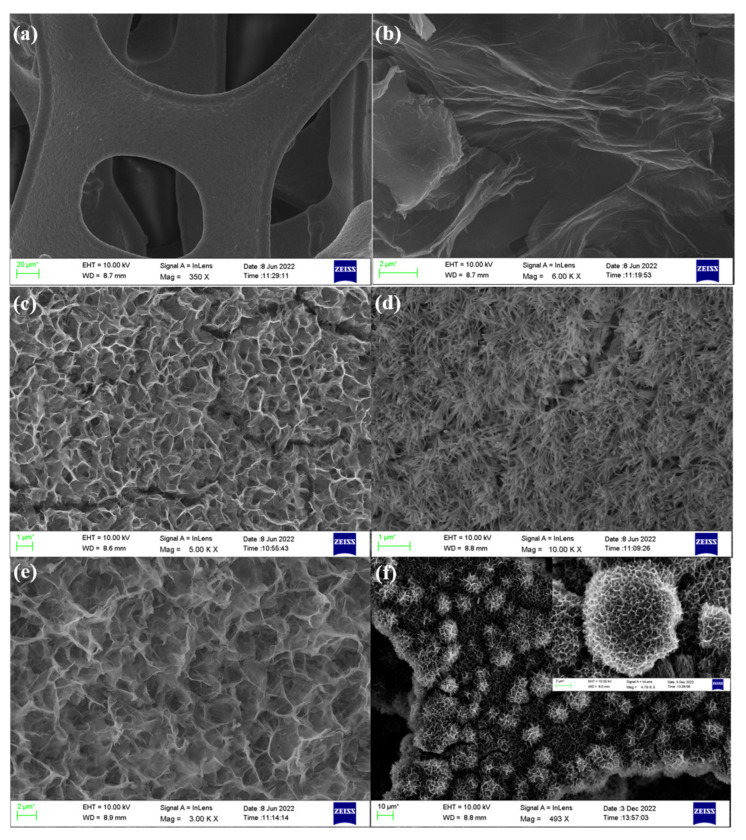
SEM images of (**a**) bare NF, (**b**) rGO/NF, (**c**) Nd_2_O_3_/rGO/NF, (**d**) Co_3_O_4_/rGO/NF, (**e**) Nd_2_O_3_/Co_3_O_4_/NF, and (**f**) Nd_2_O_3_/Co_3_O_4_/rGO/NF; the inset in (**f**) is the plot of Nd_2_O_3_/Co_3_O_4_/rGO/NF magnification.

Scanning electron microscopy was utilized to investigate the prepared electrode in order to describe the surface morphology and structure in greater detail. Figure 4a–f shows the bare NF, rGO/NF, Nd_2_O_3_/rGO/NF, Co_3_O_4_/rGO/NF, Nd_2_O_3_/Co_3_O_4_/NF, and Nd_2_O_3_/Co_3_O_4_/rGO/NF SEM images. Figure 4a makes it evident that the treated NF with a smooth surface simultaneously conveys a three-dimensional skeleton structure. After being loaded with rGO, the NF surface that had previously displayed a smooth state becomes rough, and it can be seen that visible ripple-like folds, as well as a significant number of cracks, emerge on the material surface [29], as shown in Figure 4b.In this instance, the rough surface helps with ion diffusion and transport, which significantly lowers ion transport resistance and speeds up ion movement. As illustrated in Figure 4c, cracks can be visible. On the NF, the monometallic Nd_2_O_3_ forms a network of crossing and tightly connected nanofibers, and these dense networks are less conducive to the entry of electrolytes during the electrode reaction, limiting the interaction between the electrolyte and active sites. Additionally, as demonstrated in Figure 4d, the monometallic Co_3_O_4_ results in a significant amount of nanoneedle-like structures on NF. The scanning electron microscopy image of Nd_2_O_3_/Co_3_O_4_/NF is shown in Figure 4e, and it can be seen that there are many 3D hierarchical honeycomb-like structures. The honeycomb-like structures create a sparse mesh with greater pore sizes and larger specific surface areas between each other. These honeycomb-like structures are formed of many nanowires and nanofibers together, and they have the propensity to expand upward. Nd_2_O_3_ and Co_3_O_4_ are deposited on NF during hydrothermal synthesis, creating porous spherical honeycomb-like structures when graphene is added to the composite. These 3D hierarchical spherical honeycomb structures as shown in Figure 4f facilitate the contact between the electrode material and the electrolyte solution and increase the specific surface area of the electrode material, which raises the active sites, lowers the ion transport resistance, and shortens the ion diffusion path [30].

**Figure 5:**
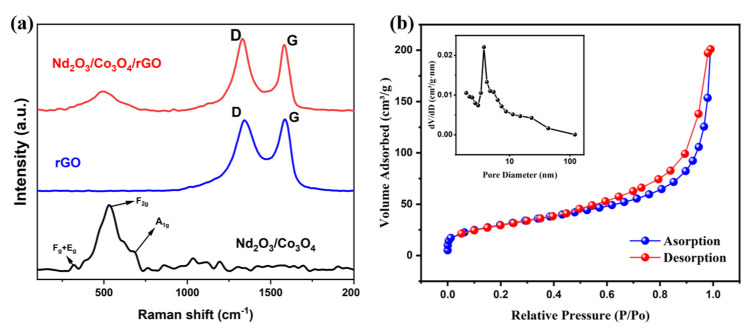
(**a**) Raman spectra of Nd_2_O_3_/Co_3_O_4_/rGO/NF; (**b**)Nitrogen adsorption and desorption isotherm of Nd_2_O_3_/Co_3_O_4_/rGO with the pore size distribution in the insets.

By comparing the Raman curves of the materials Nd_2_O_3_/Co_3_O_4_/rGO, Nd_2_O_3_/Co_3_O_4_ and GO, Raman spectroscopy is able to qualitatively determine if GO has been successfully reduced in the material. The Raman spectra of GO, Nd_2_O_3_/Co_3_O_4_/NF, and Nd_2_O_3_/Co_3_O_4_/rGO/NF electrode materials are depicted in Figure 5a. Both GO and Nd_2_O_3_/Co_3_O_4_/rGO/NF have Raman spectra that show the G-band at 1582 cm^−1^ and the D-band at 1344 cm^−1^, which are formed by the stretching vibrations of sp^2^- and sp^3^-hybridized carbon atoms in graphite, respectively [31,32]. It is customary to utilize the ratio of D-band to G-band peak intensities (I_D_/I_G_) to indicate the structural flaws in graphene and the extent of graphene reduction. Graphene structural flaws and the level of graphene reduction are frequently represented by the ratio of D-band to G-band peak intensities (I_D_/I_G_) [33]. In general, a higher I_D_/I_G_ ratio indicates a larger structural flaw in the material, in this experiment, it is indicated that a larger reduction in the GO, which was effectively transformed to rGO. Nd_2_O_3_/Co_3_O_4_/rGO has a higher I_D_/I_G_ value than rGO (0.98), which is 1.14. The Raman spectra of Nd_2_O_3_/Co_3_O_4_ and Nd_2_O_3_/Co_3_O_4_/rGO also show the presence of two additional vibrational modes [34], designated as F_2g_ and A_1g_, respectively. The band at 502 cm^−1^ corresponds to the F_2g_ of Co_3_O_4_, whereas the weak spectral band of A_1g_ at 651 cm^−1^ is assumed to be connected to the properties of the octahedral lattice positions inhabited by Co^3+^. As indicated in Figure 5a, the moderately intense F_g_ + E_g_ Raman band at 320.5 cm^−1^ was also discovered. In conclusion, the Nd_2_O_3_/Co_3_O_4_/NF electrode materials were successfully prepared, as evidenced by the Raman characteristic peaks of both Nd_2_O_3_ and Co_3_O_4_ visible in the Nd_2_O_3_/Co_3_O_4_/NF material. The aforementioned phenomena and results also support this conclusion. Due to the porous honeycomb spherical structure of the manufactured Nd_2_O_3_/Co_3_O_4_/rGO material, it is assumed to have a large specific surface area. Thus, the nitrogen adsorption-desorption method was applied to analyze the material’s properties in order to determine the specific surface area and pore size distribution. As depicted in Figure 5b, a noticeable H3-type hysteresis loop appears on the curve at higher relative pressure, reflecting a classic IV-type isotherm feature [35]. According to the test results, the Nd_2_O_3_/Co_3_O_4_/rGO composite has a large specific surface area of 107.6246 m^2^/g, which makes it advantageous to yield abundant redox sites for Faraday energy storage when redox processes occur. The Nd_2_O_3_/Co_3_O_4_/rGO material is rich in mesopores, with mesopore size largely concentrated between 3 nm–4 nm, as illustrated by the inset, which reveals the pore size distribution with an average pore size of about 12 nm.

**Figure 6:**
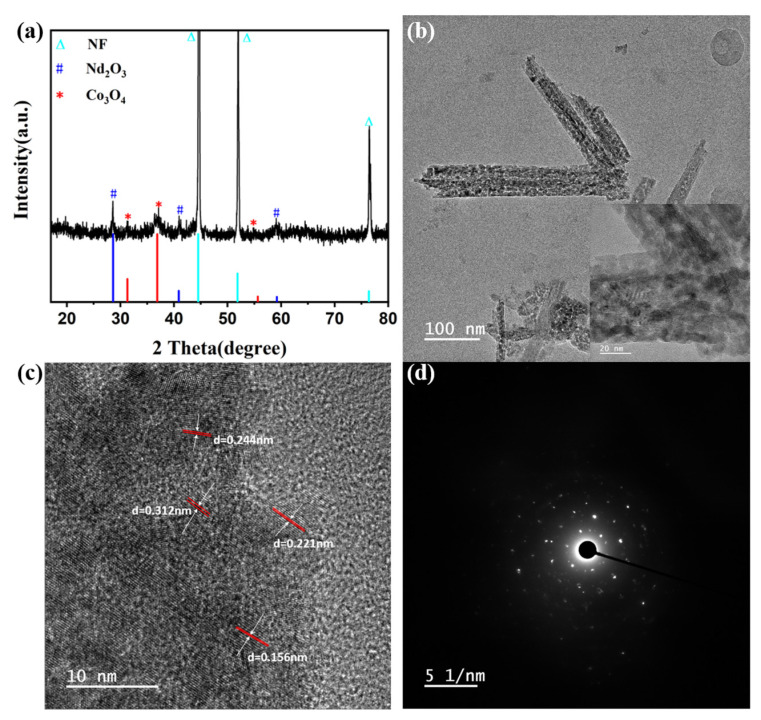
(**a**) TEM of Nd_2_O_3_/Co_3_O_4_/rGO/NF; the inset of (**a**) is the magnified view of nanosheet; (**b**) HRTEM image; (**c**) SEAD pattern of Nd_2_O_3_/Co_3_O_4_/rGO/NF and (**d**) XRD of Nd_2_O_3_/Co_3_O_4_/rGO/NF.

The constituent phases of Nd_2_O_3_/Co_3_O_4_/rGO/NF were characterized by XRDas shown in Figure 6a. The characteristic peaks of metallic nickel, which correspond to the (111), (200), and (220) crystal planes of metallic nickel, are positioned at 44.3°, 51.7°, and 76.2°, respectively. These predominant peaks are easily noticeable in the image (JCPDS card No. 04-0850). Additionally, the (220), (311), and (422) crystallographic planes of Co_3_O_4_ are represented by 31.3°, 36.9°, and 55.66° in the curve respectively, going to describe the forming of cubic-Co_3_O_4_(JCPDS card No. 42-1467) [36]. The typical Nd_2_O_3_ peaks at 2θequal to 28.6°, 40.9°, and 59.2° in Figure 6a all correspond to the (110), (200), and (220) crystal planes of cubic-Nd_2_O_3_(JCPDS card No. 83-1356) and the decreased strength of the characteristic Nd_2_O_3_ peak is thought to be due to the smaller grain size.The absence of the characteristic GO peaks in the spectrum may be induced by the effective separation of GO nanosheets by Nd_2_O_3_ and Co_3_O_4_, which leadsto highly disordered dispersion in Nd_2_O_3_/Co_3_O_4_/rGO/NF composites and increased specific capacitance [37].

The morphological structure of Nd_2_O_3_/Co_3_O_4_/rGO/NF was further inspected and demonstrated as shown in Figure 6 using transmission electron microscopy (TEM), selected area electron diffraction (SAED), and high-resolution transmission electron microscopy (HRTEM). Figure 6b clearly shows a spherical honeycomb-like structure composed of a large number of nanoparticles, which is consistent with the results presented by porous nanospheres in SEM images. With channels for their diffusion and a spherical honeycomb-like structure, the material electron and ion transport rates are greatly increased, which speeds up the redox reaction during charging and discharging. As a result of measuring HRTEM pictures of Nd_2_O_3_/Co_3_O_4_/rGO/NFas shown in Figure 6c, distinct lattice stripes with lattice spacings of 0.221 nm, 0.156 nm, and 0.312 nm, which correspond to the (200), (220), and (110) crystal planes of Nd_2_O_3_(JCPDS card No. 83-1356),were obtained. The (311) crystal plane of Co_3_O_4_(JCPDS card No. 42-1467) is represented by the lattice spacing of d = 0.4 nm. This is consistent with the XRD test results that were reported. As further evidence of the material polycrystalline structures, Figure 6d also displays a selected area electron diffraction pattern, which displays a sequence of concentric diffraction rings.

### 3.2. Electrochemical Properties

**Figure 7:**
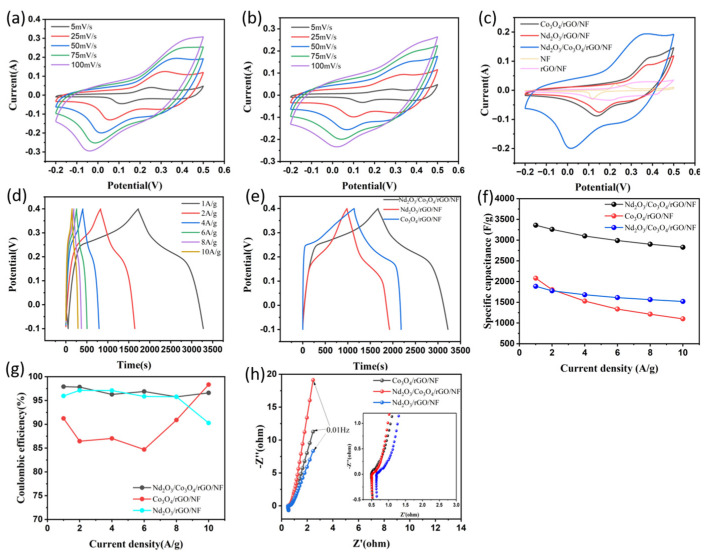
(**a**,**b**) CV curves of Nd_2_O_3_/Co_3_O_4_/rGO/NF and Nd_2_O_3_/Co_3_O_4_/NF electrodes under different scan rates; (**c**) comparison CV curves of Nd_2_O_3_/Co_3_O_4_/rGO/NF, Nd_2_O_3_/rGO/NF, Co_3_O_4_/rGO/NF, rGO/NF and NF electrodes at 50 mV/s; (**d**) GCD curves of Nd_2_O_3_/Co_3_O4/rGO/NF electrodes at different current densities; (**e**) comparison GCD curves of Nd_2_O_3_/Co_3_O_4_/rGO/NF, Nd_2_O_3_/rGO/NF and Co_3_O_4_/rGO/NF electrodes at 1 A/g; (**f**) specific capacitances of Nd_2_O_3_/Co_3_O_4_/rGO/NF, Nd_2_O_3_/rGO/NF and Co_3_O_4_/rGO/NF electrodes at different current densities; (**g**) the Coulombic efficiencies of Nd_2_O_3_/Co_3_O_4_/rGO/NF, Nd_2_O_3_/rGO/NF and Co_3_O_4_/rGO/NF at different current densities; (**h**) the Nyquist plots of Nd_2_O_3_/Co_3_O_4_/rGO/NF, Nd_2_O_3_/rGO/NF and Co_3_O_4_/rGO/NF electrodes, the circuit inset is the high-frequency region. All of the electrochemical measurements described above were performed in a 6 mol/L KOH solution.

The cyclic voltammetry, constant current charge/discharge, and electrochemical impedance spectroscopy at an electrochemical workstation are used to examine the electrochemical performance of the produced electrode Nd_2_O_3_/Co_3_O_4_/rGOfurther. All electrochemical characterizations in the typical three-electrode test are carried out with the test ambient environment being a 6 mol/L KOH solution.As demonstrated in Figure 7a, the CV images of the Nd_2_O_3_/Co_3_O_4_/rGO/NF electrode at 0.7 V (−0.2 V to −0.5 V) at various scan rates (5 to 100 mV/s) are also depicted. The value of the electrode-specific capacity can be calculated by integrating the CV curve as shown in Figure 7a according to Equation (1).When the scan rates are 5, 25, 50, 75, and 100mV/s, the magnitudes of the specific capacity of the corresponding Nd_2_O_3_/Co_3_O_4_/rGO/NF electrodes are 2272.2, 1550.6, 1185.1, 961.2, and 800.5C/g, respectively.

The existence of redox peaks in the scan curves can still be seen clearly at a scan rate of 100 mV/s, and redox peaks can also be seen in all CV curves. This event demonstrates that the electrode material is employed to store charge through a Faraday redox reaction, which in turn supports and demonstrates the material effective use as a pseudocapacitor [38] (the magnitude of the specific capacitance is proportional to the current density). The CV curves of Nd_2_O_3_/Co_3_O_4_/rGO/NF, Nd_2_O_3_/rGO/NF, Co_3_O_4_/rGO/NF, rGO/NF and NF are displayed in Figure 7c at a scan rate of 50 mV/s. In contrast, it is clear that Nd_2_O_3_/Co_3_O_4_/rGO/NF has a larger integral area of the CV curve than do Nd_2_O_3_/rGO/NF and Co_3_O_4_/rGO/NF, proving that the material has a higher specific capacitance.The rGO/NF CV curves are subrectangular generally and do not exhibit more pronounced redox peaks, which are indicative of double-layer capacitance properties. Comparatively, the area created by the CV curve is found to be substantially bigger than that of NF, showing that the reduced graphene oxide also provides a small amount of capacitance.As Figure 7a demonstrated, the general shape of the CV curve did not change as the scan rate increased, and it continued to exhibit a fast and reversible redox property, demonstrating that the electrode material can achieve a rapid exchange of electrons and ions on its surface and has good stability. Due to the neutralization reaction of some electrolytes during the reaction and the internal resistance brought on by the polarization of the electrode material itself, the oxidation peak tends to be greater and the reduction peak tends to be smaller as the scan rate rises [39]. As can be seen from Figure 7b, the redox peak of the Nd_2_O_3_/Co_3_O_4_/NF electrode is lower than that of the Nd_2_O_3_/Co_3_O_4_/rGO/NF electrode. This, together with the latter morphological composition, suggests that the 3D hierarchical porous spherical honeycomb shape created by the addition of graphene is more conducive to the interaction between the ions in the electrolyte and the electrode material, increasing the active sites and thus accelerating the Faraday redox rate. The electrode material electrochemical properties are drastically improved further. The ion transfer reaction between Nd_2_O_3_ and Co_3_O_4_ in a solution of 6 mol/LKOH is described in the following for the CV test [40].

Co_3_O_4_ + OH^−^ + H_2_O ⇌3CoOOH + e^−^ (5)

CoOOH + OH^−^⇌CoO_2_ + H_2_O + e^−^ (6)

Nd_2_O_3_ + H_2_O ⇌2NdOOH (7)

NdOOH + OH^−^⇌Nd(OH)_2_ + e^−^ (8)

As a result, the valence change of Nd and Co is what is responsible for the redox peaks in the CV curves [41]. Figure 7d displays the constant-current charge/discharge curves of the Nd_2_O_3_/Co_3_O_4_/rGO/NF electrode at various current densities with a voltage window of −0.1 to 0.4 V in order to further describe the electrochemical characteristics of the electrode materials. It can also be obtained that only a very small portion of the capacitance of the electrode material is provided by the double-layer since rGO sets up a double-layer capacitance in the electrode material that causes the potential to change accordingly with time but almost flat with the potential axis [42]. This phenomenon demonstrates the pseudo-capacitance of the electrode [43]. When the current density drops to 1A/g, the plateau duration is the longest and the charge/discharge time is also the longest. In the charge/discharge curves, it is noticeable that the slope of the potential versus time is different but there is always a plateau area. The GCD comparison curves of the electrodes consisting of Nd_2_O_3_/Co_3_O_4_/rGO/NF, Co_3_O_4_/rGO/NF, and Nd_2_O_3_/rGO/NF are shown in Figure 7e when the current density is 1 A/g. The following equation provides the qualitative GCD curve computation of the electrode-specific capacitance [44].

(9) $$C=\frac{\left(I\;\times \Delta t\right)}{\left(m\;\times \Delta V\right)}$$

When the current densities are 1, 2, 4, 6, 8, and 10 A/g, respectively, the magnitudes of the specific capacitance of the corresponding Nd_2_O_3_/Co_3_O_4_/rGO/NF electrodes are 3359.6, 3259.6, 3098.4, 2989.2, 2900.8, and 2828.0 F/g, respectively. The magnitudes of the specific capacitance of the Co_3_O_4_/rGO/NF electrodes are 2082, 1804.8, 1528.8, 1335.6, 1212.8, and 1100.0 F/g, respectively. The specific capacitance values of the Nd_2_O_3_/rGO/NF electrodes were 1886, 1776.4, 1681.6, 1612.8, 1563.2, and 1520.0 F/g as shown in Figure 7f. It has been demonstrated that when the current density becomeshigher, the rate of redox processes occurring on the electrode surface likewise accelerates, and the diffusion rate prevents the loss of ions. As a result, as the current density rises, the specific capacitance of the electrode diminishes [45]. The maximum specific capacitance of the Nd_2_O_3_/Co_3_O_4_/rGO/NF electrode may reach 3359.6 F/g when the current density is 1 A/g, which is 1.61 times more than that of the monometallic Co_3_O_4_/rGO/NF electrode (2082 F/g) and 1.78 times greater than that of the monometallic Nd_2_O_3_/rGO/NF electrode (1886 F/g). These results solidify the great ability of the Nd_2_O_3_/Co_3_O_4_/rGO/NF composite electrode to optimize electrode structure, increase electrode-specific capacitance size, and enhance electrochemical performance. The monometallic CV and GCD curves of the Co_3_O_4_/rGO/NF and Nd_2_O_3_/rGO/NF electrodes are depicted in Figure S2. The combination of specific capacitance and Coulomb efficiency at various current densities determines whether this porous spherical electrode material can have remarkable application prospects in the SC area. The specific capacitance of the Nd_2_O_3_/Co_3_O_4_/rGO/NF electrode may still acquire 2828.0 F/g when the current density goes up to 10 A/g, as shown in Figure 7f. Despite the decrease in current density, the two electrodes of the symmetric SC composed of this electrode are still able to maintain a high Coulomb efficiency as shown in Figure 7g. The following equation may be employed to identify the electrode Coulomb efficiency [46].

(10) $$\eta =\frac{t_{d}}{t_{c}}\times 100\%$$

where the discharge and charging times, respectively, are denoted by t_d_ (s) and t_c_ (s) respectively.

This discloses the exceptional reversibility of charging and discharging that this reactive pseudocapacitive material possesses, confirming its excellent electrochemical performance and highlighting the enormous potential of this electrode material for use in the SC area.

Using the electrochemical impedance spectroscopy (EIS) technique, impedance investigations were carried out in a 6 mol/L KOH solution at an open circuit potential from 100 kHz to 0.01 Hz in order to identify the impedance magnitude of the Nd_2_O_3_/Co_3_O_4_/rGO/NF electrode. The principal factors that influence the electrode material resistance, according to the fitted equivalent circuit, are the charge transfer resistance (R_ct_) and diffusion resistance (Warburg impedance). The beginning internal resistance (R_s_), which can be derived from the *x*-axis intercept [47], is mainly composed of the ionic resistance of the electrolyte solution, the intrinsic resistance of the active material, and the contact resistance at the interface between the active material and collector. The R_ct_ decreases as the semicircle becomes more restricted. Smaller Warburg impedances result from steeper slopes. The collected Nyquist curves were compared and analyzed, as shown in Figure 7h. The impedance of the Nd_2_O_3_/Co_3_O_4_/rGO/NF electrode increases sharply and more steeply than that of the other electrodes in the low-frequency region [48], inferring a smaller Warburg resistance, which implies a lower electrolyte diffusion resistance and a higher ion diffusion rate of the electrolyte in this electrode material [49]. In the high-frequency region, the R_s_ values for Nd_2_O_3_/Co_3_O_4_/rGO/NF, Co_3_O_4_/rGO/NF, and Nd_2_O_3_/rGO/NF are 0.49, 0.65, and 0.52 Ω, respectively. The diameter of the semicircle in the high-frequency region represents the R_ct_,and the absence of a large semicircle there denotes a very rapid charge transfer rate, which can be related to the fact that the 3D hierarchical porous spherical honeycomb-like structures made of nanoparticles are exceedingly porous. While increasing the permeability of the electrolyte, making it easier to penetrate the electroactive material, and narrowing the ion/electron diffusion path [50]. The shape of a porous spherical honeycomb composed of nanosheets with a high specific surface area offers more electroactive sites for rapid diffusion and movement of ions. By creating a special microsphere-like structure in the area between the nanosheets, which also increases electrical conductivity and electrochemical activity, the space between the nanosheets slows the volume expansion brought on by cyclic charging and discharging.

**Figure 8:**
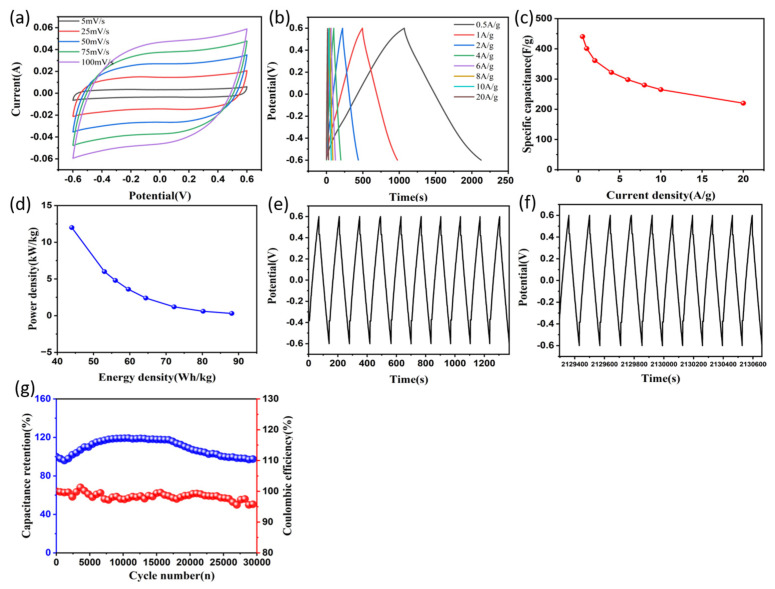
(**a**) CV curves of the symmetric SC under different scan rates; (**b**) GCD curves of the SC at different current densities; (**c**) specific capacitances of the SC at different current densities; (**d**) Ragone plot related to power and energy densities of the SC; (**e**,**f**) the GCD curves for the first 10 cycles and the last 10 cycles out of 30,000 cycles; (**g**) the capacitance retention and Coulombic efficiency at a current density of 10 A/g for 30,000 cycles; The electrolyte for all the above electrochemical tests was 6 mol/L KOH solution.

The experiments were carried out under the conditions of employing Nd_2_O_3_/Co_3_O_4_/rGO/NF electrodes as the positive and negative electrodes, filter paper as the septum between the two electrodes, and KOH solution with a concentration of 6 mol/L as the test environment for the assembled symmetrical SC in order to further examine the application prospects of Nd_2_O_3_/Co_3_O_4_/rGO/NF electrode materials as supercapacitor electrode materials. Figure 8a depicts the CV curves of the SC while the scan rate (5~100 mV/s) was altered. Based on observation and comparison, it can be inferred that the curves at various scan rates all had a roughly rectangular shape, indicating the good pseudocapacitive performance of the electrode material [51], and that the overall shape did not vary as the scan rate was changed, demonstrating the high rate performance of the SC and the great design. When the scan rate is altered, the overall shape retains the same, evidencing the high rate performance and practical properties of the SC [52]. The GCD curves of the matching SCs are shown in Figure 8b when the current density is altered. All of the GCD curves exhibit prominent triangles, indicating that the Nd_2_O_3_/Co_3_O_4_/rGO/NF electrode is in a condition of rapid ion transport. With the change in current density, the shapes of the triangles do not change considerably, indicating that the SC has high capacitive performance [53]. When the current densities are 0.5, 1, 2, 4, 6, 8, 10, and 20 A/g, the corresponding specific capacitances of 440.4, 400.8, 361, 322, 298, 280, 265,220 F/g are shown in Figure 8c. According to Equations (2) and (3), Figure 8d depicts the link between the energy density and power density of the SC. 30,000 cycles of charge and discharge at a current density of 10 A/g and a voltage window of −0.6~0.6V were measured on the symmetric SC made up of Nd_2_O_3_/Co_3_O_4_/rGO/NF electrodes. The capacitor GCD curves for the first 10 cycles are shown in Figure 8e, and the GCD curves for the last 10 cycles as shown in Figure 8f demonstrate that the reaction is still reversible. After 30,000 cycles at a high current density, the capacitance retention rate was still 95.7% as shown in Figure 8g. The stability of the composite electrode material made of Nd_2_O_3_/Co_3_O_4_/rGO is confirmed by this finding. Through the images, a slight rise in specific capacitance was seen during cycling, which was thought to be caused by the electrode material being activated throughout the constant charging and discharging process, exposing additional active sites. In the efficient cycling stability performance of the super-electricity, the synergistic implications of Nd_2_O_3_, Co_3_O_4_, and rGO play a key role. The development of in situ voids in the 3D hierarchical porous spherical honeycomb-like structures created on the NF substrate significantly boosts the effective active area in contact with the electrolyte, effectively reduces volume expansion brought on by frequent charging and discharging, and strengthens cycling performance.

**Figure 9:**
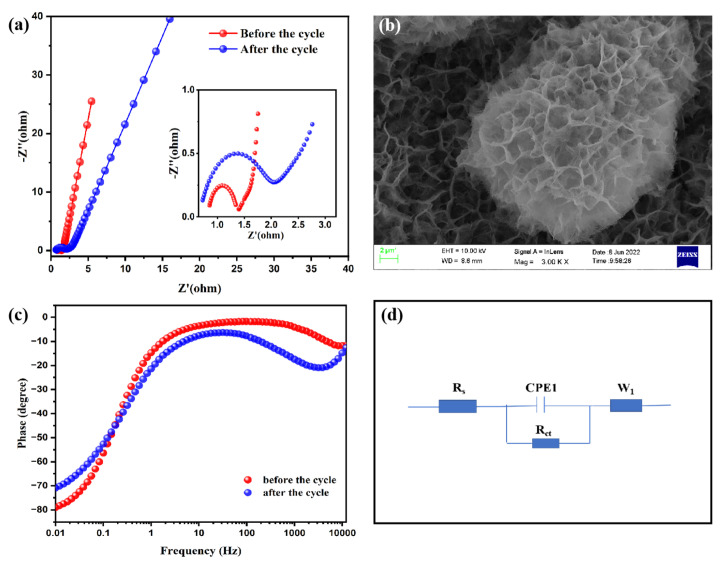
(**a**) the Nyquist plot of the symmetric SC before and after 30,000 cycles, the inset is the plot in the high-frequency region; (**b**) SEM image of the symmetric SC after discharging/charging for 30,000 cycles at a current density of 10 A/g. The electrolyte was 6 mol/L KOH solution; (**c**) Bode phase diagram of the symmetric SC before and after 30,000 cycles; (**d**) the simulated supercapacitor equivalent circuit diagram.

Electrochemical impedance spectroscopy (EIS) was employed to verify the materials’ properties before and after 30,000 cycles. Figure 9a shows the SC Nyquist curves before and after cycling. With anR_ct_ value of 1.1 Ω, which is higher than that of the pre-cycled material (R_ct_ = 0.5 Ω), the post-cycled material displays a wider radius of arc in the high-frequency band, indicating an increase in charge transfer resistance. Additionally, the slope is lower in the low-frequency range, suggesting a rise in Warburg impedance. All resistance magnitudes, however, are raised properly. After 30,000 cycles at a current density of 10 A/g, Figure 9b displays the SEM pictures of the 3D hierarchical spherical honeycomb-like structures of Nd_2_O_3_/Co_3_O_4_/rGO/NF. The morphology and structure of the electrode do not change significantly during repeated charging and discharging, with the exception of a small amount of volume expansion and a slight reduction in surface roughness brought on by the redox reaction. The Nd_2_O_3_/Co_3_O_4_/rGO/NF electrode may have strong structural stability as a result of this.The Bode phase frequency plots of the supercapacitor before and after cycling are shown in Figure 9c. It can be seen from the curves that the after-cycling curve is a little bit lower than the before-cycling curve, and both phase angles tend to stabilize and approach 0° at the high frequency area. At 100 Hz, the phase angle in the low- and medium-frequency zone starts to progressively grow, and at the limit frequency of 0.01 Hz, the phase angles are −79° and −70.9°, respectively, both exhibiting good capacitance characteristics. The simulated supercapacitor equivalent circuit diagram is shown in Figure 9d. Table 1 displays that the constructed Nd_2_O_3_/Co_3_O_4_/rGO/NF electrode has greater performance in all respects when assessing the materials employed in this work with those employed in earlier reports.

## 4. Conclusions

The stable composite material of Nd_2_O_3_, Co_3_O_4,_ and reduced graphene oxide with unique 3D hierarchical porous spherical honeycomb-like structures on nickel foam was successfully prepared using a straightforward one-step hydrothermal synthesis technique coupled with a high-temperature annealing process. The data indicated that the metallic oxides cubic-Nd_2_O_3_ and cubic-Co_3_O_4_ efficiently inhibit the aggregation and accumulation of reduced graphene oxide throughout the reaction process, while the specific surface area of Nd_2_O_3_/Co_3_O_4_/rGO material up to 107.6246 m^2^/g with an average pore size of about 12 nm improves the interaction area between the porous microspheres and the electrolyte. Along with its outstanding electrical conductivity, reduced graphene oxide enhances dual capacitance performance in addition to its contribution to electron transport, thereby supplying more energy to the reaction. The Nd_2_O_3_/Co_3_O_4_/rGO electrode in the experiment had a specific capacitance of 3359.6 F/g at 1A/g in a 6 M KOH solution. The composite electrodes were assembled into symmetrical supercapacitors and tested electrochemically, the results were remarkable. The capacitance retention rate of 95.7% even at a high current density of 10 A/g after 30,000 continuous charge/discharge cycles, and the specific capacitance reached a maximum of 440.4 F/g at a capacitance density of 0.5 A/g. As well, the energy density is 88.1 Wh/kg at a power density of 300 W/kg. In addition to deriving from the particular porous honeycomb spherical structure of Nd_2_O_3_/Co_3_O_4_/rGO, the composite electrode’s high electrochemical performance in the three-electrode and two-electrode systems is also directly tied to the synergistic impact between the three. This opens up new opportunities and avenues for the development of rare earth metal oxide Nd_2_O_3_ as a new kind of Faraday energy storage supercapacitor electrode material.

## Figures and Tables

**Table 1:** Comparison of electrochemical performances of Nd_2_O_3_/Co_3_O_4_/rGO/NF composite electrode in our work with previous reports.

Electrode Materials	Specific Capacitance	Cycling Performance	Energy Density at Power Density	Ref.
Co_3_O_4_/rGO composites	1152 F/g at 1 A/g	89.1 % (5000 cycles)	57.26 Wh/kg at 2240.68 W/kg	[54]
CeO_2_/Co_3_O_4_ composites	255 F/g at 0.25 A/g	90.1 % (3000 cycles)	27.5 Wh/kg at 500 W/kg	[55]
Nd(OH)_3_/g-C_3_N_4_composites	426.40 F/g at 1A/g	88.3% (3000 cycles)	no data	[56]
Fe_3_O_4_/C composites	490.2 F/g at 1A/g	90.7 % (1000 cycles)	34.3 Wh/kg at 1003.9 W/kg	[57]
Nd_2_O_3_/Co_3_O_4_/rGO/NF	3359.6 F/g at 1A/g	95.7% (30,000 cycles)	88.1 Wh/kg at 300 W/kg	This work

## Data Availability

Not applicable.

## Supplementary Materials

The following supporting information can be downloaded at: https://www.mdpi.com/article/10.3390/ma16041694/s1, S1: Preparation of Nd_2_O_3_/Co_3_O_4_/NF composite electrode; S2: Preparation of Nd_2_O_3_/rGO/NF composite electrode; S3: Preparation of Co_3_O_4_/rGO/NF composite electrode; S4: Preparation of rGO/NF composite electrode; Figure S1: EDS elemental mapping images of the composite (Nd, Co, Ni, O and C); Figure S2: (a,b) CV curves of Co_3_O_4_/rGO/NFand Nd_2_O_3_/rGO/NF electrodes under different scan rates; (c,d) GCD curves of Co_3_O_4_/rGO/NFand Nd_2_O_3_/rGO/NF electrodes at different current densities.
