# Supplementary material for: Fabrication of 3D HierarchicalSphericalHoneycomb-Like Nd2O3/Co3O4/Graphene/Nickel Foam Composite Electrode Material for High-Performance Supercapacitors
Source: Materials (Basel). 2023 Feb 17;16(4):1694. doi: 10.3390/ma16041694 (PMC9963774; doi:10.3390/ma16041694)
Supplement: Supplementary file 1 [file materials-16-01694-s001.zip › materials-2182801-supplementary.pdf]

Nd_2O_3/Co_3O_4/graphene/nickel foam composite electrode material for high-performance supercapacitors

Huihui Liang[a], Shasha Wang[a], Shixiang Lu[a,*], Wenguo Xu[a], Min Zhou[b]

S1 Preparation of Nd_2O_3/Co_3O_4/NF composite electrode

0.6 mmol (0.175 g) $Co(NO_3)_2 \cdot 6H_2O$, 0.6 mmol (0.247 g) $Nd(CH_3COO)_3 \cdot 5H_2O$ and 1 mmol (0.060 g) urea were dissolved in 15 mL of deionized water and stirred magnetically for 40 min to form a homogeneous solution. A portion of the above-treated NF and mixed solution was transferred to an autoclave lined with polytetrafluoroethylene and reacted for 12 h in an oven at 140 °C. After being washed with ethanol and deionized water, the Nd_2O_3/Co_3O_4 precursor was dried in an oven at 90 °C for 4 h. After 2 h of annealing in a muffle furnace at 250 °C, the Nd_2O_3/Co_3O_4/NF composite electrode was produced.

S2 Preparation of Nd_2O_3/rGO/NF composite electrode

In 7.5 mL of deionized water, 0.6 mmol (0.247 g) $Nd(CH_3COO)_3 \cdot 5H_2O$ and 1 mmol (0.060 g) urea were dissolved and magnetically agitated for 40 min to generate a homogenous solution. Then, 7.5 mL of homogenous graphene oxide suspension (2 mg/mL) was added. Continue magnetic stirring for 40 min to ensure thorough mixing. The processed NF and combined solution were placed in an autoclave with polytetrafluoroethylene and reacted for 12 h in an oven at 140 °C. The precursor was cleaned with ethanol and deionized water before being dried in a 90 °C oven for 4 h. Finally, the Nd_2O_3/rGO/NF composite electrode was created by annealing it for 2 h in a muffle furnace at 250 °C.

S3 Preparation of Co_3O_4/rGO/NF composite electrode

0.6 mmol (0.175 g) $Co(NO_3)_2·6H_2O$ and 1 mmol (0.060 g) urea were diluted in 7.5 mL deionized water and magnetically agitated for 40 min to generate a homogenous solution. Then 7.5 mL of homogenous graphene oxide suspension (2 mg/mL, ultrasonication for 2 h) was added. Continue magnetic stirring for 40 min to thoroughly combine. A portion of the above-mentioned treated NF and the combined solution were placed to an autoclave with polytetrafluoroethylene and reacted in an oven at 140 °C for 12 h. The precursor was rinsed with ethanol and deionized water before being dried in an oven at 90 °C for 4 h. Finally, the Co_3O_4/rGO/NF composite electrode was formed after 2 h of annealing in a muffle furnace at 250 °C.

S4 Preparation of rGO/NF composite electrode

7.5 mL of deionized water and 7.5 mL of 2 mg/mL graphene oxide suspension were added to the beaker, mixed well with magnetic stirring for 40 min. A portion of the above-mentioned treated NF and the combined solution were placed to an autoclave with polytetrafluoroethylene and reacted in an oven at 140 °C for 12 h. The surface of the NF was cleaned with ethanol and deionized water before being dried in a 90 °C oven for 4 h. Finally, the rGO/NF composite electrode was formed after 2 h of annealing in a muffle furnace at 250 °C.

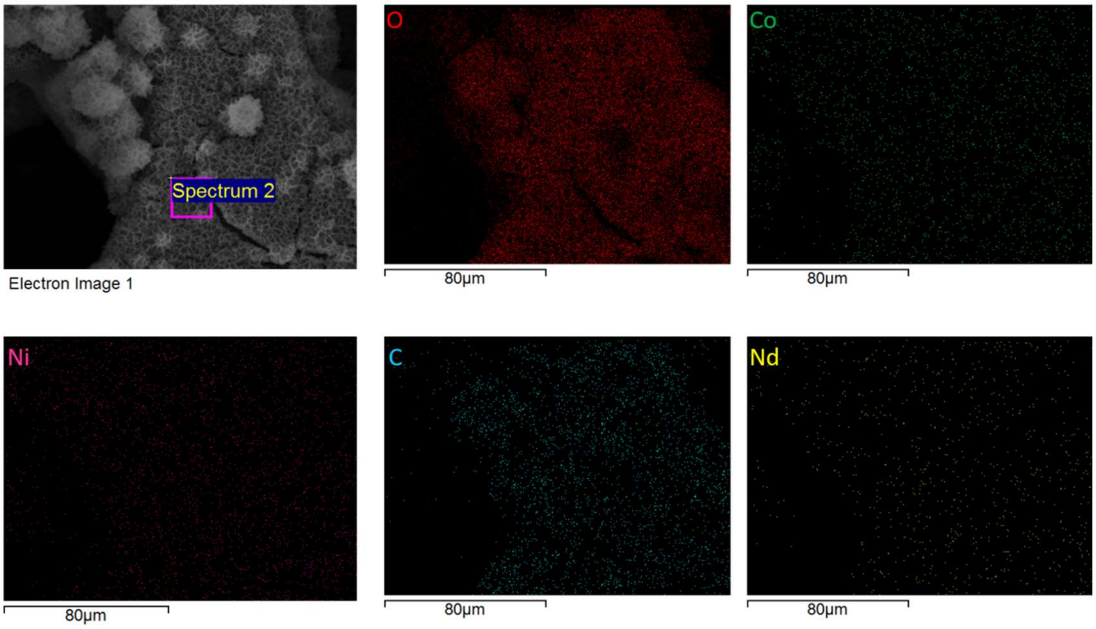

Figure S1. EDS elemental mapping images of the composite (Nd, Co, Ni, O and C).

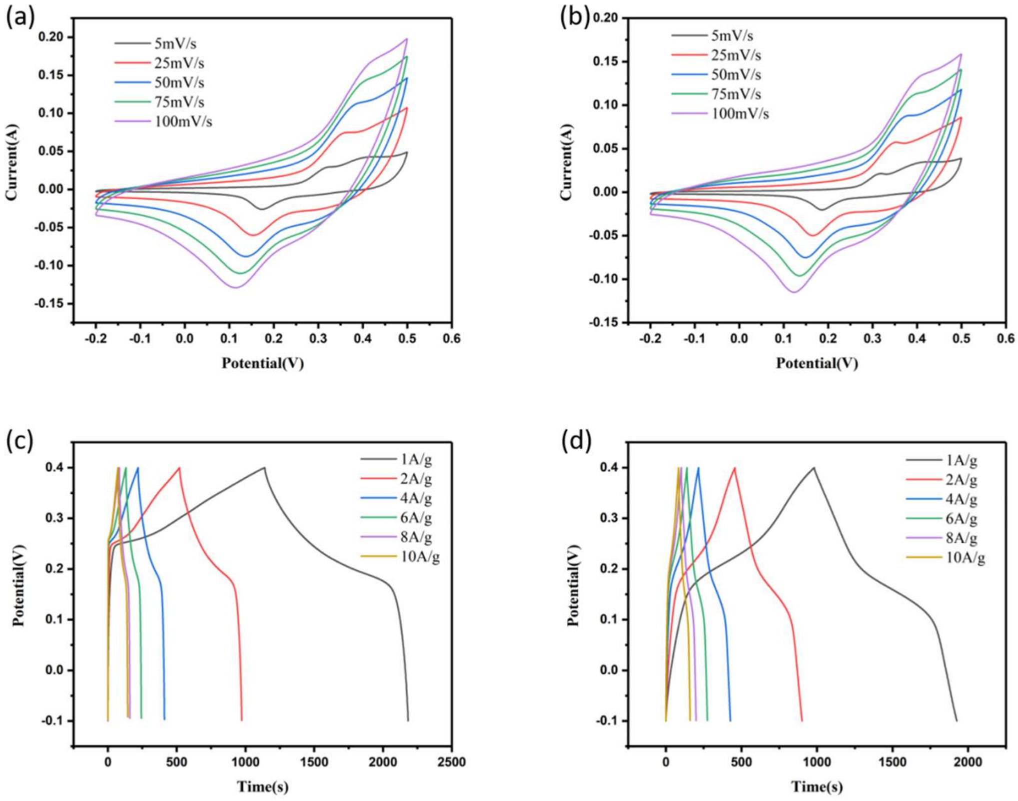

Figure S2 (a, b) CV curves of Co_3O_4/rGO/NF and Nd_2O_3/rGO/NF electrodes under different scan rates; (c, d) GCD curves of Co_3O_4/rGO/NF and Nd_2O_3/rGO/NF electrodes at different current densities.
